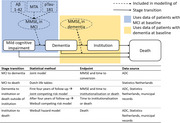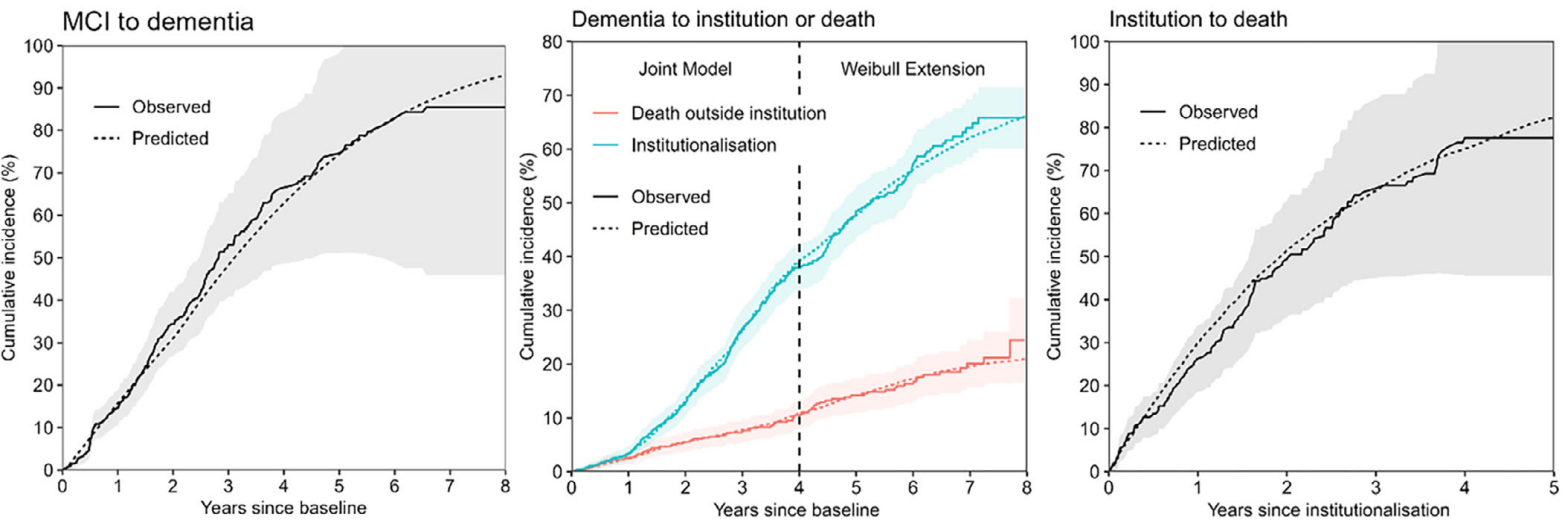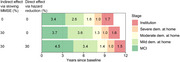# Development of a simulation model of Alzheimer's Disease to evaluate disease modifying therapies in a memory clinic population

**DOI:** 10.1002/alz70860_104261

**Published:** 2025-12-23

**Authors:** Pieter J. van der Veere, Hana M. Broulíková, Jeroen Hoogland, Ron Handels, Everard G.B. Vijverberg, Argonde C. van Harten, Charlotte E. Teunissen, Frederik Barkhof, Elsmarieke van de Giessen, Wiesje M. van der Flier, Johannes Berkhof

**Affiliations:** ^1^ Amsterdam Neuroscience, Neurodegeneration, Amsterdam, Netherlands; ^2^ Alzheimer Center Amsterdam, Neurology, Vrije Universiteit Amsterdam, Amsterdam UMC location VUmc, Amsterdam, Netherlands; ^3^ Department of Epidemiology and Data Science, Amsterdam UMC, Amsterdam, Netherlands; ^4^ Department of Health Sciences, Faculty of Science, Vrije Universiteit Amsterdam, Amsterdam, Netherlands; ^5^ Ministry of Health of the Czech Republic, Prague, Czech Republic; ^6^ Alzheimer Center Limburg, Mental Health and Neuroscience Research Institute, Maastricht University, Maastricht, Netherlands; ^7^ Karolinska Institutet, Dept for Neurobiology, Care Sciences and Society, Center for Alzheimer Research, Div of neurogeriatrics, Solna, Sweden; ^8^ Amsterdam Neuroscience, Neurodegeneration, Amsterdam, Noord‐Holland, Netherlands; ^9^ Neurochemistry Laboratory, Department of Clinical Chemistry, Amsterdam UMC, location VUmc, Amsterdam, Netherlands; ^10^ Neurochemistry Laboratory, Department of Clinical Chemistry, Amsterdam Neuroscience, Vrije Universiteit Amsterdam, Amsterdam UMC, Amsterdam, Netherlands; ^11^ Amsterdam Neuroscience, Brain Imaging, Amsterdam, Netherlands; ^12^ Queen Square Institute of Neurology and Centre for Medical Image Computing, University College London, London, Greater London, United Kingdom; ^13^ Department of Radiology and Nuclear Medicine, Vrije Universiteit Amsterdam, Amsterdam University Medical Center, location VUmc, Amsterdam, Netherlands; ^14^ Department of Radiology and Nuclear Medicine, Amsterdam UMC, Vrije Universiteit Amsterdam, Amsterdam Neuroscience, Amsterdam, Netherlands; ^15^ Alzheimer Center, Department of Neurology, Amsterdam UMC, Vrije Universiteit Amsterdam, Amsterdam Neuroscience, Amsterdam, Netherlands

## Abstract

**Background:**

Simulation models can extrapolate short‐term treatment effects observed in clinical trials of amyloid‐targeting therapies over longer disease trajectories. Current simulation models include clinically diagnosed AD patients without biomarker confirmation, whereas only biomarker‐confirmed patients are eligible for treatment. We aim to develop a model for the simulation of biomarker‐confirmed AD memory clinic patients natural disease trajectories and evaluate time gained with different hypothetical treatment scenario.

**Method:**

We developed an AD microsimulation model with stages mild cognitive impairment (MCI), mild, moderate, and severe community dementia, institutionalisation, and death. We estimated the model with data from 388 amyloid‐positive MCI and 762 amyloid‐positive dementia patients from the Amsterdam Dementia Cohort. Longitudinal follow‐up information included assessments of mini‐mental state examination (MMSE), time to dementia (*n* = 219), time to institutionalisation (*n* = 277) and time to death (*n* = 262). Two joint models formed the core of the simulation model describing MMSE decline and transition from MCI to dementia, and MMSE decline and the transition from dementia to institutionalisation or death (Figure 1). Community dementia stages were defined by MMSE: >20 (mild), 10‐20 (moderate), and <10 (severe). Model performance was assessed by visually comparing observed and predicted cumulative incidence curves. We performed a microsimulation of the trajectory of 1000 MCI patients (66±7yr, 47%F, MMSE 26±2) to determine the mean time per stage.

**Result:**

The predicted cumulative incidences were similar to the observed cumulative incidences, indicating good model performance (Figure 2). The mean natural time per stage was: 3.4yrs (MCI), 2.6yrs (mild), 1.6yrs (moderate), 1.0yrs (severe community dementia), and 1.7yrs (institution); totalling 10.2yrs from MCI to death. An intervention slowing MMSE decline by 30% during MCI and mild dementia increased MCI duration by 4mths, increased mild dementia duration by 12mths, and delayed institutionalisation by 12mths (Figure 3). An additional direct intervention effect reducing the hazard of transitioning from MCI or mild dementia by 30%, further extended MCI duration by 10mths, while mild dementia duration increased by 10mnths instead of 12mnths.

**Conclusion:**

We developed a simulation model to evaluate amyloid‐targeting therapies in MCI and mild dementia. The model can help quantify the effect of different treatment mechanisms and longitudinal consequences of treatment scenarios.